# Can height categories replace weight categories in striking martial arts competitions? A pilot study

**DOI:** 10.1515/hukin-2015-0065

**Published:** 2015-10-14

**Authors:** Gal Dubnov-Raz, Yael Mashiach-Arazi, Ariella Nouriel, Raanan Raz, Naama W. Constantini

**Affiliations:** 1 Exercise, Nutrition and Lifestyle Clinic, The Edmond and Lily Safra Children’s Hospital, Sheba Medical Center, Tel Hashomer, Israel.; 2 Sackler Faculty of Medicine, Tel Aviv University, Tel Aviv, Israel.; 3 School of Nutritional Sciences, The Robert H. Smith Faculty of Agriculture, Food and Environment, The Hebrew University of Jerusalem, Rehovot, Israel.; 4 Sport Medicine Center, Department of Orthopedic Surgery, Hadassah- Hebrew University Medical Center, Jerusalem, Israel.

**Keywords:** karate, eating disorders, rapid weight loss, athletes

## Abstract

In most combat sports and martial arts, athletes compete within weight categories. Disordered eating behaviors and intentional pre-competition rapid weight loss are commonly seen in this population, attributed to weight categorization. We examined if height categories can be used as an alternative to weight categories for competition, in order to protect the health of athletes.

Height and weight of 169 child and adolescent competitive karate athletes were measured. Participants were divided into eleven hypothetical weight categories of 5 kg increments, and eleven hypothetical height categories of 5 cm increments. We calculated the coefficient of variation of height and weight by each division method. We also calculated how many participants fit into corresponding categories of both height and weight, and how many would shift a category if divided by height.

There was a high correlation between height and weight (r = 0.91, p<0.001). The mean range of heights seen within current weight categories was reduced by 83% when participants were divided by height. When allocating athletes by height categories, 74% of athletes would shift up or down one weight category at most, compared with the current categorization method.

We conclude that dividing young karate athletes by height categories significantly reduced the range of heights of competitors within the category. Such categorization would not cause athletes to compete against much heavier opponents in most cases. Using height categories as a means to reduce eating disorders in combat sports should be further examined.

## Introduction

In most types of combat sports and martial arts, namely judo, wrestling, boxing, taekwondo, karate and others, athletes compete against their opponents within a specific weight category. Competitors usually aspire to be among the heaviest in their designated weight category, as a possible physical and tactical advantage over lighter opponents. These athletes often weigh more than the upper limit for their weight category during training. An intentional rapid weight loss before competitions is a common practice in such sports, occurring in an estimated 60–90% of athletes (Franchini et al., 2012). Pre-competition rapid weight loss frequently begins in adolescence, and continues throughout the competitive career : ~60% of judo athletes started such rapid weight reduction at the ages of 12–15 years, whereas Brazilian karate and taekwondo athletes reported beginning these procedures at the ages of 13.6 ± 1.4 and 14.2 ± 2.1 years, respectively (Franchini et al., 2012). In our study including 75 competitive adolescent Taekwondo athletes, the youngest age of the first rapid weight loss attempt was 10 years (Constantini et al., 2011; Dubnov-Raz et al, 2015).

The negative effects of rapid weight loss on sports performance and athletes’ health have been thoroughly studied (Franchini et al., 2012; Fogelholm, 1994; Mendes et al., 2013). Such behavior could significantly decrease lean body mass (Kukidome et al., 2008; Sagayama et al., 2014), hinder performance (Hall and Lane, 2001), reduce fluid and carbohydrate stores during competition (Pettersson and Berg, 2014a; Pettersson and Berg, 2014b), impair immunity (Shimizu et al., 2011; Tsai et al., 2011), decrease vigor (Degoutte et al., 2006; Filaire et al., 2001; Hall and Lane, 2001), and increase fatigue, anger, anxiety, tension and confusion (Degoutte et al., 2006; Filaire et al., 2001; Hall and Lane, 2001; Steen and Brownell, 1990). The most tragic consequence of such behavior documented was the death of three wrestlers that attempted to rapidly lose weight by dehydration prior to competition (Centers for Disease Control and Prevention, 1998).

Abnormal dieting behavior by athletes in weight-category sports is not limited to pre-competition, but might also occur throughout the training season. A high rate of disordered eating behaviors in such athletes from both sexes has been frequently reported (Fogelholm and Hiilloskorpi, 1999; Sundgot-Borgen and Torstveit, 2004). The most probable reason for this situation is the constant need to remain within a specific weight range, in which athletes are only a few kilograms higher than the upper limit for their designated weight category.

In order to protect the health of athletes in weight class sports, Mountjoy (2008) from the International Olympic Committee Medical Commission called upon sport governing bodies to carefully evaluate their rules in order to decrease the health risks to their elite athletes. She also recommended changes in methods of weigh-ins in weight class sports, again, for the purpose of decreasing health risks. Finding an alternative to categorizing athletes for competition by body weight is certainly justified from the medical perspective.

It is important to differentiate full-contact types of fights such as boxing, wrestling, judo, full-contact karate, jiu-jitsu, mixed-martial arts and others, from striking martial arts, such as the classical Shotokan karate and taekwondo. In full-contact fighting sports, fighting occurs at a relatively close distance between competitors, and includes punching, grabbing, holding and/or throwing, depending on specific rules and regulations in each type of sport. Hence, both body height and mass may be advantageous for competitors in such sports. In striking martial art fights, however, competitors usually stand relatively far from each other, and rapidly move with forward striking movements, kicks, and punches, in a manner somewhat similar in motion to fencing. This intuitively suggests that limb length may be a more important factor for scoring than body weight. Therefore, for tactical reasons in striking martial arts, height may play a more significant role than weight.

Taken together, it seems that the use of weight categories for competition poses a significant health risk, and at least for striking martial arts athletes, body weight may have less significance than body height taking into consideration the tactical aspect. In search for a safer way to categorize athletes in martial arts, yet while maintaining contestant equality in body size, we hypothesized that height categories may be an alternative to weight categories in such sports.

The aims of this study were to examine the relationship between height and weight in competitive karate fighters in the pediatric age range, where weight and height have a large variability, and to examine if height categories could be used as an alternative to weight categories.

## Material and Methods

### Participants

The study population included 169 male and female competitive karate athletes that competed in the Israeli National Karate Championship for children and adolescents in July 2009. A representative from the research team approached all competitors on the competition day as they entered the competition hall for weigh-ins, explained the study details, answered questions, and obtained verbal consent from the competitor, as well as written consent from the accompanying parent to participate in the study. The study was approved by the Institutional Review Board of the Sheba Medical Center, Tel Hashomer, Israel and was conducted according to the Declaration of Helsinki.

### Measures - demographic and anthropometric data

Height was measured to the nearest 0.1 cm by a wall-mounted stadiometer (SECA 206, SECA gmbh, Hamburg, Germany) with the children barefoot and with their joined heels, buttocks, shoulders, and head touching the wall. Body weight was measured to the nearest 0.1 kg by an 8-electrode bio-impedance analyzer (BC-418, Tanita Corporation, Tokyo, Japan).

### Procedures-creating height and weight categories

Similarly to other combat sports, karate is a martial art in which athletes are commonly categorized for competitions according to age, sex, and weight, in increments of about 5 kg (e.g., International Karate Union, 2014). After all study data were collected, participants were divided into eleven hypothetical weight categories of increments of 5 kg, from under 25 kg to 70 kg and above, according to the study participants measured weight range. Participants were also divided into eleven hypothetical height categories of 5 cm increments, according to their actual measured heights, that ranged from under 120 cm to 165 cm and above. Height and weight categories were numbered from 1 (lowest) to 11 (highest).

### Statistical analysis

Continuous variables are expressed as mean ± standard deviation. The Pearson’s correlation coefficient was used to measure the relationship between participant’s height and weight. In order to examine the relationships between the created height and weight categories, we divided all participants into categories according to both their measured height and weight, and then calculated how many fit into corresponding categories of both height and weight (e.g., belong to category 1 (lowest) in both height (<120 cm) and weight (<25 kg), or to category 2 in both height and weight (120–125 cm and 25–30 kg), etc.). We also calculated the number of participants in each height category that belonged to non-corresponding weight categories (e.g., belong to height category 3, but *not* to weight category 3). Afterwards, the percentage of athletes who would remain in their “original” category (i.e., corresponding weight and height) if divided by height was calculated, as well as the percentage of athletes that “moved” at least one or more weight categories when divided by height. In order to compare height and weight variability independently of their different units of measure, we used the coefficient of variation (CV, which is calculated as the standard deviation divided by the mean). Statistical analyses were performed using IBM SPSS Statistics for Windows version 21.

## Results

**Figure 1:**
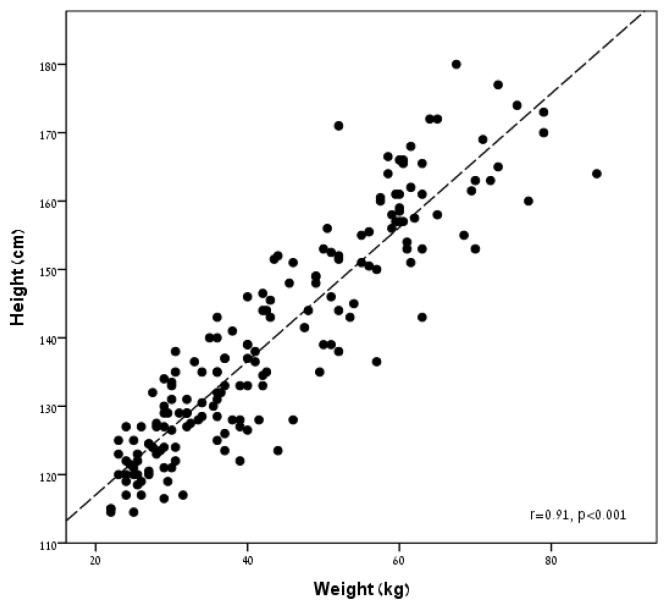
The relationship between height and weight in the study participants

Clinical and anthropometric data of the study participants are presented in Table 1. The relationship between height and weight in all study participants is presented in Figure 1. There was a strong correlation between height and weight in the whole study population (r = 0.91, p<0.001), as well as when examined separately for males (r=0.92, p<0.001) and females (r=0.88, p<0.001).

Table 2 presents the positions of all study participants according to both height and weight categories, demonstrating how many would remain in corresponding categories of both height and weight, the number of athletes that remained in the same category when divided by height, as well as how many would shift one, two, or more categories by the new categorization method. Overall, 33% of athletes remained in the same category, while a total of 74% of athletes moved at most only ±1 category following the change of categorization method.

Table 3 presents the weight and height statistics of all competitors when they were categorized for competition by weight, height, or age only. When competitors were divided by the new height categories, the mean CV of their height within a category was reduced by 83% (from 6.4% to 1.1%) in comparison to the mean CV of heights when divided by the traditional weight categories. In comparison, when using the existing weight categories, the mean CV of weights within a category was lower by 73% (from 13.8% to 3.7%) in comparison to the mean CV of weights if divided by height categories. Hence, when using height categories, there was a larger decrease in height variability than there was an increase in weight variability. When competitors were hypothetically divided based on age alone, an even greater range of both height and weight was obtained.

## Discussion

The main aim of this pilot study was to examine the novel possibility of applying height categories as an alternative to weight categories in striking martial arts. Eventually, the ultimate goal of such a change would be to reduce the high rate of disordered eating seen in combat sports, while not interfering, and perhaps even improving contestant equality in body size during competition. There was a strong correlation between athletes’ height and weight, which demonstrates their close relationship in the pediatric age range. The variation in competitor heights seen with the traditional weight categorization was reduced when participants were divided by height categories. Finally, we found that when dividing athletes by height categories, three-fourths of athletes would shift up or down only one category at most when compared to their original weight category. This means that in most cases, the ‘tradeoff’ for dividing competitors by height would be a moderate increase in the range of weights of competitors within the category - which in this type of sport may be of lower importance in combat.

Height is commonly regarded as advantageous in many sports. While merely being tall is obviously no guarantee for athletic success, tall individuals are commonly seen in sports such as high-jump, basketball, volleyball, tennis, and others. Regarding the advantage of height in martial arts, an analysis of body size of Taekwondo fighters from the Olympic games of 2000–2010, showed that in most cases and in both sexes, the winners had mean heights slightly larger, and mean BMIs slightly lower, than those of the non-winners (Kazemi et al., 2013). In a study of adult karate athletes, those competing in fights (“kumite”) were higher on average than non-fighters (competing in “kata”) (Koropanovski et al., 2011). Therefore, it seems that there is a natural selection of the body type and size in striking martial arts, as in other sports. It is possible that by using height categories and removing the limitation of body weight, a different average body type will be favored in striking martial arts. Despite these reports and others that suggest an advantage to higher contestants, we are aware of very few sports in which heights are actually used for contestant grouping and equality. In Nunchaku fights, competitors are divided by 10 cm height categories, exhibiting the importance of arm length in this striking-type martial art (World Nunchaku Association, 2014). In ski jumping, the maximal ski length allowed is related to the total body height and BMI of the competitor (International Ski Federation, 2014).

The negative effects of pre-competition rapid weight loss on sports performance and athlete health are well known (Franchini et al., 2012; Fogelholm, 1994; Hall and Lane, 2001; Mendes et al., 2013). The basis for our novel concept described herein was the need to reduce the frequent and unhealthy disordered eating behaviors seen in athletes from weight-category combat sports (Mountjoy, 2008). For example, in a study of Norwegian elite athletes, 18% of males and 30% of females competing in weight-class sports fulfilled criteria for eating disorders (Sundgot-Borgen and Torstveit, 2004). In a study of Finnish athletes, 93% of males and 85% of females competing in sports with weight categories disclosed abnormal weight loss behavior (Fogelholm and Hiilloskorpi, 1999). In a study of US male collegiate athletes, those from weight-class sports were more likely to be in the disordered eating group than those who practiced endurance and ball game sports; they were also more likely to binge eat, exercise excessively, and diet or fast (Chatterton and Petrie, 2013). In our study of 75 competitive adolescent Taekwondo athletes, we found that 40% of the rapid-weight losers lost over 3% of their body weight (Constantini et al., 2011) which is an amount considered to pose a health risk in these circumstances. Interestingly, rapid weight loss was self-assessed to cause performance decline in 20% of athletes, by hindering motivation, technique, power, body composition and mood (Constantini et al., 2011). By finding an alternative to weight-class categorization, we are certain that there could be a marked reduction in the frequent preoccupation of these athletes with their body weight and the unhealthy and dangerous behaviors associated with it. In addition, it may even result in better athletic performance in competition and increased fairness in competitor body size.

We acknowledge that our study has several limitations. Regarding generalizability, we measured children and adolescents only, therefore our results cannot be extrapolated to adults without further research. We focused on a striking-type martial art, in which body mass may be of less tactical importance than in other combat sports, such as judo and wrestling. We acknowledge that in real-life tournaments, competitors are divided not only by weight, but also by sex and age groups. Our limited sample size restricted us from performing more complex, sex- and age-specific analyses. From a medical point of view, it still remains to be seen whether the use of height categories can indeed reduce disordered eating behaviors, as this was the major rational for this study. Finally, our concept of height categories remains to be examined ‘in real life’ by contest managers and karate/taekwondo organizations, and tested in actual large-scale tournaments.

In summary, this study was motivated by the high rate of disordered eating frequently seen in sports with weight-categories, and examined a novel alternative of using height categories in striking martial arts. We showed that young competitive karate athletes can be divided by height categories for competition, with only a moderate increase in the range of weights of competitors within the category. The use of height categories in weight-class sports, and perhaps in other types of sport, should be further examined. Such categorization can potentially improve tactical aspects and equality in body size among athletes, and most importantly, serve as a means to reduce disordered eating behaviors and improve athletes’ health. Therefore, we suggest that at least for the striking martial arts such as karate and taekwondo, the use of height categories for competition should be further examined.

## Figures and Tables

**Table 1:** Clinical and anthropometric data of study participants

Characteristic	All study participants (n=169)
Males, n (%)	129 (76%)
Age, years	
mean ± SD	11.4±2.6
range	17–8
Height, cm	
mean ± SD	139±16.1
range	180–114.5
Weight, kg	
mean ± SD	42.3±14.8
range	86–22
BMI, kg/m2	
mean ± SD	21.4±3.6
range	14.7–32.0

SD - standard deviation; BMI - body mass index

**Table 2:** The number of study participants according to each height and weight category

Weight (kg)		1	2	3	4	5	6	7	8	9	10	11	
												
Height (cm)		<25	25–29.9	30–34.9	35–39.9	40–44.9	45–49.9	50–54.9	55–59.9	60–64.9	65–69.9	70+	Total
1	<120	5	6	1	0	0	0	0	0	0	0	0	12
2	120–124.9	5	15	3	2	1	0	0	0	0	0	0	26
3	125–129.9	2	7	8	6	2	1	0	0	0	0	0	26
4	130–134.9	0	3	5	6	3	0	0	0	0	0	0	17
5	135–139.9	0	0	4	4	6	1	3	1	0	0	0	19
6	140–144.9	0	0	0	4	3	2	2	0	1	0	0	12
7	145–149.9	0	0	0	0	3	4	2	0	0	0	0	9
8	150–154.9	0	0	0	0	2	1	4	3	4	0	1	15
9	155–159.9	0	0	0	0	0	0	0	3	4	2	0	9
10	160–164.9	0	0	0	0	0	0	0	3	3	0	3	9
11	165+	0	0	0	0	0	0	1	1	6	2	5	15
	Total	12	31	21	22	20	9	12	11	18	4	9	

**Table 3:** Height and weight statistics of study participants when divided into categories of weight (5 kg increments), height (5 cm increments), or age (1 year increments). Data presented are the means of weight and height range and standard deviation of all 11 categories

	Weight categories	Height categories	Age categories
Weight			
Mean range (kg)	5.0	20.9	28.6
Mean standard deviation (kg)	1.7	6.2	8.4
Mean coefficient of variation (%)	3.7	13.8	18.8
Height			
Mean range (cm)	24.2	4.6	35.2
Mean standard deviation (cm)	7.1	1.6	7.2
Mean coefficient of variation (%)	6.4	1.1	5.0
